# Neural Basis of Intrinsic Motivation: Evidence from Event-Related Potentials

**DOI:** 10.1155/2015/698725

**Published:** 2015-09-27

**Authors:** Jia Jin, Liping Yu, Qingguo Ma

**Affiliations:** ^1^Business School, Ningbo University, Ningbo 315211, China; ^2^School of Management, Zhejiang University, Hangzhou 310027, China; ^3^Neuromanagement Lab, Zhejiang University, Hangzhou 310027, China

## Abstract

Human intrinsic motivation is of great importance in human behavior. However, although researchers have focused on this topic for decades, its neural basis was still unclear. The current study employed event-related potentials to investigate the neural disparity between an interesting stop-watch (SW) task and a boring watch-stop task (WS) to understand the neural mechanisms of intrinsic motivation. Our data showed that, in the cue priming stage, the cue of the SW task elicited smaller N2 amplitude than that of the WS task. Furthermore, in the outcome feedback stage, the outcome of the SW task induced smaller FRN amplitude and larger P300 amplitude than that of the WS task. These results suggested that human intrinsic motivation did exist and that it can be detected at the neural level. Furthermore, intrinsic motivation could be quantitatively indexed by the amplitude of ERP components, such as N2, FRN, and P300, in the cue priming stage or feedback stage. Quantitative measurements would also be convenient for intrinsic motivation to be added as a candidate social factor in the construction of a machine learning model.

## 1. Introduction

Human intrinsic motivation is concerned by both academic research and practical application for its great significance for human behavior. Therefore, it has gained considerable attention from scientists and educationalist for decades. However, for the intrinsic motivation it was always difficult to be directly measured and observed, and its explicit impact on human behavior was unclear which went against learning human's behavior.

In recent years, the rapid development of the neuroscience techniques made it possible for us to open the “black box” of our brain and observe people's neural responses directly. For the study of intrinsic motivation, researchers also considered if it was possible to probe it at brain level. Quirin et al. [1] employed functional magnetic resonance imaging (fMRI) to investigate the different motivation of power and affiliation, in which they found power-related versus affiliation-related social motivations had differential brain networks. In another study, Murayama et al. (2010) [2] also employed fMRI to investigate the neural evidence of interaction between intrinsic and extrinsic motivation at the spatial level. At the feedback stage, they found that BOLD signal in ventral striatum was prominently decreased when the extra reward for performance was removed at a later session of the task, while such a phenomenon was not observed in the control group where no performance-based monetary reward was provided for both sessions. These studies suggested that the variation of human intrinsic motivation could be reflected in brain activity which could not be early measured at behavior level.

In addition to fMRI, event-related potential (ERP) was another widely used neuroscience tool which can make up the temporal dynamic accuracy of fMRI. Therefore, in the current study we employed ERPs to compare the neural discrepancy of the two tasks with different levels of intrinsic fun throughout the whole task process. The purpose of our study was to explore the electrophysiological dynamics of the human intrinsic motivation through EEG recordings. According to previous study about ERPs and motivation, we supposed that three ERP components would appear in the current experiment, N2 in the cue priming stage and FRN and P300 in the feedback stage.

The negative component N2 was reported peaking around 200 to 300 ms after the onset of a stimulus [3, 4]. Previous studies found that the visual N2 component was related to the deviation from the perception of the target cognitive control and action inhibition processes [4]. For example, Eimer (1993) [5] employed the go/no-go paradigm and conducted two experiments, in which the participants were asked to respond to a letter (go stimulus) but not to another (no-go stimulus). Results showed that the N2 enhancement elicited by the no-go stimuli was larger than that induced by the go stimuli, which reached its maximum in frontal areas. They suggested that this was because of the response mismatch and action inhibition. Subsequent studies also explained the larger nontarget N2 amplitude as subjects' inhibition of an anticipated response to the target [6].

FRN was another candidate component which would be found in the current study. It was reported in various tasks that FRN was related to the affective/motivation at the feedback stage [7, 8]. In an early study conducted by Gehring and Willoughby (2002) [8], they found a prominent differentiated FRN (d-FRN) toward the divergence of the loss gain feedback, which was suggested to reflect the subjective motivational and affective evaluation of the revealed outcome. Additionally, further studies also confirmed that the evaluative process indexed by FRN is sensitive to the motivational significance of ongoing event [7, 9–11]. For instance, in Ma et al.'s [10] 2014 work, they found that high effort could induce larger differentiated FRN responses to the reward and nonreward discrepancy across two experimental conditions. They suggested that this was because effort might increase subjective evaluation toward subsequent reward which was reflected in the FRN amplitude deflection.

In the outcome feedback stage, there always appeared another important ERP component, P300, which was always examined accompanying FRN. P300 was a positive ERP component peaking around 200–600 ms after the onset of feedback [12]. It was reported that P300 was sensitive to the magnitude [13, 14] and the valence of reward [11, 12, 15]. Furthermore, previous studies also agreed that P300 could also represent the attentional allocation and motivational/affective significance [10, 16, 17]. For instance, one of our recent studies [17] adopted a gambling task in the social context and was found independent of FRN; there was a general P300 divergence across agents of different degrees of closeness to the subjects which suggested that the valence effect of P300 could reflect motivational/affective implication of the outcome.

In the current study, in order to investigate the internal motivation of two tasks, we intended to compare electrophysiological response at the cue priming stage and feedback stage. According to the literature mentioned above, we expected that there would be a N2 component discrepancy in priming stage, in which the boring WS task would elicit a N2 enhancement compared with the interesting SW task, which suggested subjects' expectation to the interesting task. When it came to the feedback stage, we supposed that, compared with WS task, smaller FRN amplitude accompanied by larger P300 amplitude would appear in SW task, reflecting the higher subjective motivation to the interesting task's outcome.

## 2. Materials and Methods

### 2.1. Subjects

Sixteen healthy native Chinese graduate and undergraduate students (8 males), aged from 18 to 25 (mean age = 23.23; SD = 1.78) were enrolled. All of them with self-reported right-handedness and had normal or corrected-to-normal vision and did not have any history of neurological disorder or mental disease. Prior to the commencement of the experiment, informed consent was obtained from all participants. The study was also approved by the Internal Review Board of Zhejiang University Neuromanagement Lab.

### 2.2. Stimuli

The experiment included two blocks, and there were 45 trials in each block. Two tasks with different intrinsic motivation were adapted from Murayama et al.'s 2010 work [2]. In stop-watch (SW) task, the subjects were asked to stop an automatically started watch by pressing a button. They won the current trial only if the time of the watch finally fell within a specific deviation from 5 s time point. In order to make sure that the participants can succeed approximately 50% trials on average, a pilot study of thirty students was conducted before the formal experiment to confirm the time deviation. According to the result of the pilot study, the time duration of winning was determined as 70 ms deviation from 5 s time point. When it comes to the watch-stop (WS) task, the watch stopped automatically and the participants were only asked to simply press the button when it stopped. The stop timing for WS trials was varied between 4.2 and 5.8 seconds randomly, in purpose of matching the time duration of SW trials generally. There existed a 600–1000 ms randomized blank interval between trials. In each trial, a task cue was first presented for 2000 ms, indicating which task would be performed. After 600–1000 ms interval of cue onset, the task started and outcome of the performance was revealed for 2 s and interval across tasks was varied between 800 and 1200 ms. Stimuli were presented sequentially in the center of the CRT computer screen (6.2°  ×  6.2°).

### 2.3. Procedure

In a shield room participants were comfortably seated 1 m away from a computer-controlled CRT monitor. Subjects were provided with a keypad to make their responses. They were instructed to complete one of the two tasks in each trial according to the cue instruction. The formal experiment started after a pilot practice. Participants were also asked to minimize body and muscle movements during the experiment. Stimuli, recording triggers, and responses were presented and recorded using E-Prime 2.0 software package (Psychology Software Tools, Pittsburgh, PA, USA).

### 2.4. EEG Recordings and Analyses

For the data recording, EEG was recorded with an electrode elastic cap with 64 Ag/AgCl electrodes according to the standard international 10–20 system and Neuroscan Synamp2 Amplifier (Scan 4.3.1, Neurosoft Labs, Inc., Virginia, USA). The sampling rate was 500 Hz and with band-pass 0.05–70 Hz. A frontal electrode site between FPz and Fz was used for ground and left mastoid was chosen for reference. Electrooculogram (EOG) was also recorded from electrodes placed at 10 mm from the lateral canthi of both eyes (horizontal EOG) as well as above and below the left eye (vertical EOG). The experiment started when the electrode impedances were maintained below 5 kΩ.

For the data analysis, Neuroscan 4.5 software was used. The EOG artifacts were corrected offline for all subjects during preprocessing, which were corrected using the method initially proposed by Semlitsch et al. (1986) [18]. Trials containing amplifier clipping, bursts of electromyography activity, or peak-to-peak deflection exceeding ±100 *μ*V were excluded from final analysis. Data was then transferred to the average of the left and right mastoids reference offline. ERPs were digitally filtered with a low pass filter at 30 Hz (24 dB/octave).

The EEG recordings were segmented for the epoch from 200 ms before the onset of target to 800 ms after the onset. The first pretarget of 200 ms was regarded as the baseline. In cue stage analysis, data was collapsed based on the two kinds of task cues. Based on visual observation of grand-average waveforms and previous ERP guidelines of Picton et al. (2000) [19], N2 component was analyzed. According to the scalp distribution of N2 and the previous studies [20, 21], we chose time range of 270–350 ms and selected nine electrode sites, namely, F1, Fz, F2, FC1, FCz, FC2, C1, Cz, and C2, in frontal and central areas for statistical analysis. Repeated measure ANOVAs were conducted to examine the effect of N2 difference of the two task cues.

For the analysis of outcome feedback, there were three conditions, WS feedback and winning and failing results in SW task. Based on visual observation of grand-average waveforms and previous ERP reports on outcome feedback [7, 9], two ERP components, FRN and P300, were analyzed. According to the scalp distribution of FRN and the previous studies [7, 8], we chose time range of 160–200 ms and selected nine electrode sites, namely, F1,Fz, F2, FC1, FCz, FC2, C1, Cz, and C2, in frontal and central areas where it elicited the largest FRN amplitude, for statistical analysis. Similarly, we chose time window of 250–350 and nine electrode sites C1, Cz, C2, CP1, CPz, CP2, P1, Pz, and P2 for the analysis of P300. Similar repeated measure ANOVAs were also conducted for FRN and P300. The Greenhouse-Geisser [22] correction was applied in all statistical analyses when necessary (uncorrected* df* are reported with the *ε* and corrected *P* values), and the Bonferroni correction was used for multiple paired comparisons.

## 3. Results

**Figure 1:**
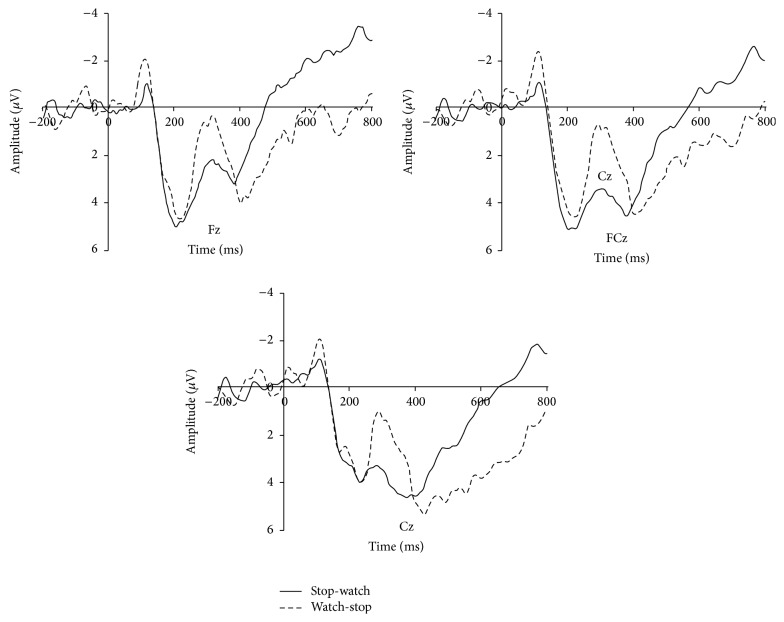
N2 results. For illustrative purpose, grand-average ERP waveforms of N2 from three frontal midline electrodes (Fz, FCz, and Cz) were plotted as a function of conditions.

As shown in Figure 1, repeated measure ANOVA results of N2 revealed significant main effect of cue category (*F*(1,15) = 6.252, *P* = 0.024, *η* ^2^ = 0.294) while the main effect of electrodes (*F*(8,120) = 2.200, *P* = 0.093, *ε* = 0.419) and interaction effect of cue and electrodes were not observed (*F*(8,120) < 1). The mean amplitude of N2 showed cue of WS task (mean = 1.374 *μ*V, SD = 1.256) elicited a larger N2 (negative polarity, smaller voltage means larger amplitude) amplitude than that of SW task (mean = 3.212 *μ*V, SD = 1.154).

**Figure 2:**
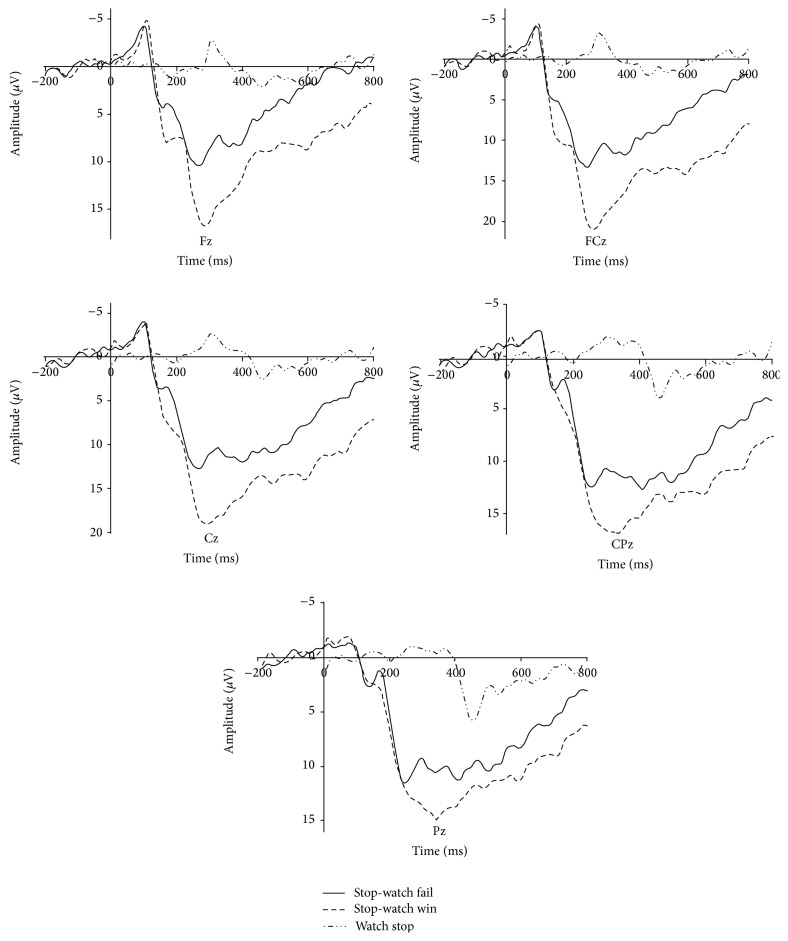
FRN and P300 results. For illustrative purpose, grand-average ERP waveforms of FRN from three frontal midline electrodes (Fz, FCz, and Cz) and P300 from two parietal electrodes (Cz, CPz, and Pz) were plotted as a function of conditions.

The general waveform of outcome feedback was shown in Figure 2. Repeated measure ANOVA results of FRN showed significant main effect of outcome valence (*F*(2, 30) = 38.938, *P* = 0.000, *η* ^2^ = 0.722). Pairwise *t*-test showed that the winning trials (mean = 7.916, SD = 1.004) elicited smaller FRN (negative polarity, smaller voltage means larger amplitude) amplitude than that of failing trials (*P* < 0.001, mean = 7.916, and SD = 1.004) and WS trials (*P* < 0.001, mean = 7.916, and SD = 1.004) while failing trials also showed a smaller FRN amplitude than WS trials (*P* = 0.017). On the other hand, the results of P300 also showed a similar effect. The main effect of P300 was observed (*F*(2,30) = 36.061, *P* = 0.000, *η* ^2^ = 0.706) and pairwise *t*-test also showed the winning trials (mean = 14.575, SD = 1.095) elicited larger P300 (positive polarity, larger voltage means larger amplitude) amplitude than that of failing trials (*P* < 0.001, mean = 11.216, and SD = 1.468) and WS trials (*P* < 0.001, mean = 72.896, and SD = 0.705) while loss trials also showed a larger P300 amplitude than WS trials (*P* = 0.049).

## 4. Discussion

This study was carried out to explore the temporal dynamics of human intrinsic motivation, which is an important facet of human behavior. We investigated how a particular task affects the subjects' intrinsic motivation by giving an interesting stop-watch (SW) task with intrinsic fun and a boring watch-stop (WS) task.

Our data showed a prominent N2 discrepancy between two task cues, suggesting the expectation of participants in performing the interesting SW task. According to the N2 literature mentioned, N2 amplitude represented mismatch and action inhibition. In the current study, the two tasks that the participants faced were of different intrinsic fun. Our results showed that N2 amplitude was enhanced when the upcoming task was the boring one, suggesting a mismatch between the expectation of the task and the actual presented task. Therefore, N2 may be a candidate index of intrinsic motivation. In addition, the current study also revealed that N2 can reflect not only the mismatch between target and nontarget as suggested by previous studies [4, 23, 24] but also the mismatch between the actual presented stimuli and their expected stimuli.

In the following feedback stage, we found that the outcome of the WS task induced larger FRN amplitude and smaller P300 amplitude than those of the SW task. Furthermore, failing trials in the SW task elicited larger FRN and smaller P300 amplitude than winning trials. These results indicated that subjective valuation of outcome was decreased in the WS task and was even lower than the failing feedback of the SW task, which is in accordance to previous findings that FRN and P300 could reflect subjects' affective/motivational evaluation of outcome. As no extrinsic incentives were given, the only source of human motivation came from the task itself, and people always showed higher intrinsic motivation to the interesting task. Therefore, a potential mechanism was that higher motivation led to higher affective evaluation toward outcome information. For the interesting SW task with higher intrinsic motivation, the outcome of the task was of higher affective significance, and the FRN effect decreased, accompanied by an increased P300 effect. Moreover, recent studies indicated that the FRN amplitude was positively correlated with the activation of reward-related regions in the brain, including the ventral striatum [25, 26]. Therefore, carrying out an interesting task was a reward in itself, even though no extrinsic reward was given. The participants considered the interesting task as fun, whereas they considered the boring task as a request to complete the task.

Meanwhile, a prominent effect for gain-loss discrepancy in the SW task was present. FRN deflection loomed smaller in the winning condition than in the failing condition, suggesting that winning feedback was of higher evaluation than that of the failing one. Furthermore, P300 reflected the valence effect of the stimuli. These results were in accordance with previous findings [12], which can also be explained by the subjects' higher evaluation of winning outcomes than that of failing outcomes. Previous studies always measured the intrinsic motivation at free-choice stage on behavioral level after participants finished the given task [27, 28] while the current study measured intrinsic motivation on brain level during the processing of tasks. Compared with the previous way, the current experiment considered amplitude of endogenous ERP component as an index of intrinsic motivation which was more objective and accurate.

The social attribute of humans was always less engrossed in studies of machine learning. As humans, we would be tired, interested, or not interested. These social factors can largely influence our behavior. Therefore, human motivation should be factored in when imitating human behavior. The current results revealed that the dynamic shifting of human intrinsic motivation from a task can reflect in the deflection of specific ERP components, such as N2, FRN, or P300. Therefore, in a machine learning model, components related to motivation may be a candidate factor of sociality.

To sum up, this study investigated the neural mechanism of intrinsic human motivation by comparing an interesting SW task and a boring WS task. The participants showed reduced N2 amplitude in the cue priming stage when the SW cue appeared, whereas, in the feedback stage, the feedback of SW task elicited reduced FRN amplitude and enhanced P300 amplitude. These results provide evidence for the existence of intrinsic motivation through electrophysiological activity on brain level and compared the degree of intrinsic motivation quantitatively. Quantitatively measured intrinsic motivation may also be a candidate social factor in a machine learning model.
